# University of Pennsylvania, Veterinary Department

**Published:** 1900-07

**Authors:** 


### COMMENCEMENT EXERCISES.

### UNIVERSITY OF PENNSYLVANIA, VETERINARY DEPARTMENT.

The commencement exercises of this department were held in conjunction with the other departments of the University at the American Academy of Music, Philadelphia, June 13, 1900, at 11 A. M. The graduating classes, members of faculties, trustees, guests of the corporation, honorary chaplain, orator of the day, the vice-provost, and provost marching in a procession to the academy. The exercises consisted of invocation by Rt. Rev. Ozi W. Whitaker; hymn, "Our Father in Heaven," this being followed by the commencement address by Wm. L. Prather, Esq., president of the University of Texas; singing, "Hail, Pennsylvania," followed by conferring of honorary degrees. The exercises closed by singing of the National anthem, "America," followed by benediction.

The degree of V.M.D. was conferred upon the following: Wm. Russell Andress, Philadelphia, Pa.; Ernest L. Cornman, Gladwyne, Pa.; Richard Y. Davison, Morton, Pa.; Glen W. Horner, Finksburg, Md.; Wm. Hughes, Harrisburg, Pa.; Henry N. Mayer, Pittsburg, Pa.; L. A. Nolan, Baltimore, Md.; Edgar W. Powell, Newtown Square, Pa.; B. H. Tallman, Farragut, Pa.; Thomas White, Philadelphia, Pa.; Hulbert Young, Washington, D. C.

A meeting of the Alumni Association was held in the evening at Houston Hall. Among those present were to be noted, from the older classes, alumnæ Leonard Pearson, dean; W. H. Ridge, M. E. Conard, F. H. Mackie, James Beatty, A. N. Lushington, George O. Forsythe, J. M. Mecray, who acted as toastmaster, John W. Adams, C. J. Marshall, H. D. Martien, L. A. Klein, J. D. Fitzpatrick, and the entire graduating class of 1900. The invited guests of the evening were Professor R. S. Huidekoper, founder of the veterinary department, and Dr. W. Horace Hoskins, editor of the JOURNAL.

Among the toasts responded to were those of the "History of Veterinary Medicine," by Professor R. S. Huidekoper, who, tracing the steps of advancement in America, pointedly referred to the rapid strides of progress from the inception of the first school in Boston, to those of to-day with their extended graded courses; this was followed by remarks on similar lines by Editor Hoskins, who cited many of the trying experiences of the pioneers in veterinary educational undertakings.

Dean Pearson, of the department of veterinary medicine, painted a glowing picture of the future of the veterinarian and the many avenues of employment opening up that demanded his services.

"Horse-show Troubles" were fully described and illustrated by exciting experiences related by Dr. W. H. Ridge, who had recently filled this unenviable rôle at the Philadelphia horse-show.

Dr. J. W. Adams, in treating of "Veterinary Medicine *versus* Human Medicine," strongly outlined the value of a course in veterinary medicine to those about to engage in human practice, and said he thought that there was little to be gained by the veterinarian in a human medicine course.

Dr. L. A. Nolan, of the graduating class, gave a succinct history of the many varied qualities possessed by the members of the class, and pointed out the great future for his classmates in the many directions where they seemed to excel. To one he pointed the way to success among the ladies and their feline and canine pets. To another this field was closed, as his successful operations among the lower animals too frequently found his patients dead. Another as a breeder, others in official places, assistant editors, until everyone felt that the profession was in safe hands in all directions for many years when handed over to the class of 1900.

Other responses were made, and with the splendid *menu* prepared it proved a very enjoyable occasion.

The election of officers for the ensuing year resulted in the selection of Dr. C. J. Marshall, president; E. M. Ranck, vice-president; J. D. Houldsworth, secretary-treasurer.

